# Supplementary material for: On the functional brain networks involved in tool-related action understanding
Source: Commun Biol. 2023 Nov 14;6:1163. doi: 10.1038/s42003-023-05518-2 (PMC10645930; doi:10.1038/s42003-023-05518-2)
Supplement: Supplementary file 2 — Reporting Summary [file 42003_2023_5518_MOESM2_ESM.pdf]

nature portfolio

Corresponding author(s): Giovanni Federico

Last updated by author(s): Oct 20, 2023

Reporting Summary

Nature Portfolio wishes to improve the reproducibility of the work that we publish. This form provides structure for consistency and transparency in reporting. For further information on Nature Portfolio policies, see our Editorial Policies and the Editorial Policy Checklist.

Statistics

For all statistical analyses, confirm that the following items are present in the figure legend, table legend, main text, or Methods section.

n/a	Confirmed	
☐	☒	The exact sample size (n) for each experimental group/condition, given as a discrete number and unit of measurement
☐	☒	A statement on whether measurements were taken from distinct samples or whether the same sample was measured repeatedly
☐	☒	The statistical test(s) used AND whether they are one- or two-sided<br>*Only common tests should be described solely by name; describe more complex techniques in the Methods section.*
☐	☒	A description of all covariates tested
☐	☒	A description of any assumptions or corrections, such as tests of normality and adjustment for multiple comparisons
☐	☒	A full description of the statistical parameters including central tendency (e.g. means) or other basic estimates (e.g. regression coefficient) AND variation (e.g. standard deviation) or associated estimates of uncertainty (e.g. confidence intervals)
☒	☐	For null hypothesis testing, the test statistic (e.g. F, t, r) with confidence intervals, effect sizes, degrees of freedom and P value noted<br>*Give P values as exact values whenever suitable.*
☒	☐	For Bayesian analysis, information on the choice of priors and Markov chain Monte Carlo settings
☒	☐	For hierarchical and complex designs, identification of the appropriate level for tests and full reporting of outcomes
☒	☐	Estimates of effect sizes (e.g. Cohen's d, Pearson's r), indicating how they were calculated

Our web collection on statistics for biologists contains articles on many of the points above.

Software and code

Policy information about availability of computer code

Data collection	The experimental paradigm and stimulus administration were implemented using Psychopy (2020), which is based on Python 2.
Data analysis	CONN 21a (https://web.conn-toolbox.org/) and MATLAB R2021b were used to conduct analyses of functional connectivity Magnetic Resonance Imaging (fcMRI). In addition, ad-hoc Python scripts were employed to organize the data in BIDS format and to extract event and supporting files for the purpose of conducting analyses.

For manuscripts utilizing custom algorithms or software that are central to the research but not yet described in published literature, software must be made available to editors and reviewers. We strongly encourage code deposition in a community repository (e.g. GitHub). See the Nature Portfolio guidelines for submitting code & software for further information.

Data

Policy information about availability of data

All manuscripts must include a data availability statement. This statement should provide the following information, where applicable:

- Accession codes, unique identifiers, or web links for publicly available datasets
- A description of any restrictions on data availability
- For clinical datasets or third party data, please ensure that the statement adheres to our policy

The data supporting the present study's findings are available from the corresponding author upon reasonable request.

Research involving human participants, their data, or biological material

Policy information about studies with human participants or human data. See also policy information about sex, gender (identity/presentation), and sexual orientation and race, ethnicity and racism.

Reporting on sex and gender	N/A. The results apply to both biological sexes and any gender.
Reporting on race, ethnicity, or other socially relevant groupings	N/A.
Population characteristics	See above.
Recruitment	Participants were enrolled through the use of social media.
Ethics oversight	Istituto di Ricovero e Cura a Carattere Scientifico (IRCCS) - Istituto Nazionale Tumori "Fondazione Pascale" (Naples, Italy).

Note that full information on the approval of the study protocol must also be provided in the manuscript.

Field-specific reporting

Please select the one below that is the best fit for your research. If you are not sure, read the appropriate sections before making your selection.

☐ Life sciences ☒ Behavioural & social sciences ☐ Ecological, evolutionary & environmental sciences

For a reference copy of the document with all sections, see nature.com/documents/nr-reporting-summary-flat.pdf

Behavioural & social sciences study design

All studies must disclose on these points even when the disclosure is negative.

Study description	This study used functional Magnetic Resonance Imaging (fMRI) to explore how the brain networks involved in understanding tool-related actions work. The study found that there were different patterns of brain activity depending on the task, with certain brain regions more active during semantic tasks and others more active during mechanical tasks. These results support the idea that action understanding is a combination of both semantic and mechanical knowledge. They also contribute to a growing understanding of how different brain regions may work together to integrate different types of action-related knowledge.
Research sample	Twenty participants who were all right-handed were enrolled. Among these participants, 11 were female, and the mean age was 25.7 years, with a standard deviation of 3.87. The following criteria were used to select participants: (i) they had no history of current or past alcohol/drug abuse; (ii) they had no current or past psychiatric illnesses; (iii) they had no history of brain injury, stroke, or any other significant clinical condition; and (iv) they were not currently or previously taking any psychoactive medications.
Sampling strategy	The present study employed ad-hoc Python scripts based on neurodesign to determine the optimal design and sample size to increase the generalizability of the findings and enhance the study's reliability and validity. The sample size for this study is consistent with similar studies.
Data collection	fMRI Data collection.
Timing	January 2021 - April 2022.
Data exclusions	One participant was excluded from the analyses due to the onset of a panic attack a few minutes before finishing the MRI session.
Non-participation	N/A.
Randomization	This is within-design study where participants were not allocated to experimental groups.

Reporting for specific materials, systems and methods

We require information from authors about some types of materials, experimental systems and methods used in many studies. Here, indicate whether each material, system or method listed is relevant to your study. If you are not sure if a list item applies to your research, read the appropriate section before selecting a response.

Materials & experimental systems

n/a	Involved in the study
☒	☐ Antibodies
☒	☐ Eukaryotic cell lines
☒	☐ Palaeontology and archaeology
☒	☐ Animals and other organisms
☒	☐ Clinical data
☒	☐ Dual use research of concern
☒	☐ Plants

Methods

n/a	Involved in the study
☒	☐ ChIP-seq
☒	☐ Flow cytometry
☐	☒ MRI-based neuroimaging

Magnetic resonance imaging

Experimental design

Design type

Cross-sectional within-subject design.

Design specifications

This cross-sectional study utilized a within-subject design with three experimental conditions, each designed to manipulate tool-related semantic/mechanical knowledge. The three tasks were as follows: (i) a yes-no recognition task designed to test semantic knowledge; (ii) a yes-no looking-to-use task designed to test mechanical knowledge; and (iii) a free-observation task designed as the control condition. The yes-no recognition task required participants to quickly identify whether a specific tool was present in an object-tool pair. The yes-no looking-to-use task required participants to determine whether a tool was usable on a given object. The free-observation task required participants to observe object-tool pairs without any explicit task. The tasks were slightly modified and adapted for MRI use, based on the experimental paradigm developed by Federico and Brandimonte (2020; Scientific Reports).

Behavioral performance measures

N/A.

Acquisition

Imaging type(s)

Structural and functional images.

Field strength

3 Tesla.

Sequence & imaging parameters

Blood-Oxygen Level Dependent images were recorded with T2-weighted Echo-Planar Images (EPI) acquired with the multi-band sequence. Functional images were collected as oblique-axial scans aligned with the anterior commissure–posterior commissure (AC-PC) line with the following parameters: 162 volumes per run (3 runs, one for each experimental task), 45 slices, TR/TE = 2000/21.4ms, flip angle = 90°, field of view = 240x240mm^2, slice thickness = 3mm, voxel size = 3x3x3mm^3, multiband factor= 2. Structural T1-weighted images were collected using a 3D T1-TFE sequence (180 sagittal slices, TR/TE = 8.1/3.7ms, flip angle = 8°, field of view 240x240mm^2, slice thickness = 1mm, voxel size = 1x1x1mm^3).

Area of acquisition

Whole brain.

Diffusion MRI ☐ Used ☒ Not used

Preprocessing

Preprocessing software

Pre-processing was carried out by implementing the standard CONN pre-processing pipeline, which included the following steps: (i) functional realignment and unwarp; (ii) slice-timing correction; (iii) outlier identification with ART-based scrubbing; (iv) direct segmentation and normalization; (v) 8-mm full-width at half-maximum Gaussian smoothing.

Normalization

Standard CONN pre-processing pipeline.

Normalization template

Montreal Neurological Institute (MNI) reference space.

Noise and artifact removal

CONN's default denoising pipeline, which included linear detrending, de-spiking and filtering (i.e., 0.008Hz < f < 0.09Hz).

Volume censoring

N/A.

Statistical modeling & inference

Model type and settings

Task-based functional connectivity statistics.

Effect(s) tested

Differences in functional connectivity (FC) between the experimental conditions were analyzed by contrasting each of the two experimental tasks with the control task, which acted as the baseline condition. In addition, a second ROI-to-ROI analysis investigated FC differences as a function of object-tool pairs' semantic consistency, irrespective of the experimental

condition.

Specify type of analysis: ☐ Whole brain ☒ ROI-based ☐ Both

Anatomical location(s): Multiple cortical areas of the left hemisphere were selected as regions of interest (ROI) from the CONN's default cortical atlas, i.e., the Harvard-Oxford atlas as distributed with FSL (https://fsl.fmrib.ox.ac.uk). Along with these ROIs, an ad-hoc ROI of the Area PF of the left inferior parietal cortex was generated with CONN and included in the analyses. This ROI consisted of a 5-mm spheric kernel centered on the following MNI coordinates: x = -55, y = -32, z = 35. Thus, the FC analysis included the following ten ROIs: the left PF; the pars triangularis and pars opercularis of the left Inferior Frontal Gyrus; the anterior, posterior and temporo-occipital part of the left Middle Temporal Gyrus; the anterior, posterior and temporo-occipital part of the left Inferior Temporal Gyrus; the Precuneous.

Statistic type for inference

(See Eklund et al. 2016)

The study used a generalized form of context-dependent psychophysiological interactions (gPPI) analysis to estimate effective FC for the experimental conditions (McLaren et al., 2012; NeuroImage). The gPPI-based general linear model included: (i) the psychological predictors, i.e., the task effects convolved with a canonical hemodynamic response function; (ii) the physiological predictors, i.e., the time series associated with the brain regions of interest; (iii) the interaction between the psychological and physiological predictors. Therefore, a hypothesis-driven gPPI analysis was performed to identify task-modulated changes in FC patterns covarying with the experimental conditions in the context of this study's design. For each trial of each run, the psychological predictors of the FC analysis were modelled considering only the appearance on screen of the first object-tool stimulus, i.e., from its onset to its end. In this way, the FC analysis was restricted to the participants' visual encoding of the object-tool pairs, thus mitigating the confounding effects of the response trials.

Correction

Pairwise ROI-to-ROI comparisons were corrected with False Discovery Rate. See: Benjamini, Y., & Hochberg, Y. (1995). Controlling the false discovery rate: a practical and powerful approach to multiple testing. Journal of the Royal statistical society: series B (Methodological), 57(1), 289-300.

Models & analysis

n/a	Involved in the study
☐	☒ Functional and/or effective connectivity
☒	☐ Graph analysis
☒	☐ Multivariate modeling or predictive analysis

Functional and/or effective connectivity

Generalized form of context-dependent psychophysiological interactions (gPPI). See: McLaren, D. G., Ries, M. L., Xu, G., & Johnson, S. C. (2012). A generalized form of context-dependent psychophysiological interactions (gPPI): a comparison to standard approaches. NeuroImage, 61(4), 1277–1286. https://doi.org/10.1016/j.neuroimage.2012.03.068.
